# Renunciation of the Vending of Quack Nostrums by an Apothecary at Buffalo

**Published:** 1848-07

**Authors:** 


*Renunciation of the vending of Quack Nostrums by an apothecary at Buffalo.*—Some months ago we published a communication addressed by an apothecary at St. Louis, Missouri, to the editor of one of the Medical Journals of that city, declaring his intentions to renounce, from principle, the sale of quack nostrums. By reference to the advertising sheet of this number of our Journal, it will be perceived that a similar course has been adopted by an apothecary of this city, Mr. H. O. Hayes. We hope the number of such instances will soon multiply, and ultimately the course be generally adopted by respectable apothecaries. We conceive that the plan which Mr. Hayes has resolved upon, constitutes a claim upon the profession which they are bound to regard, next to the paramount consideration that pure medicines will be dispensed with accuracy and fidelity.

